# Atypic Typhoid

**Published:** 1916-10

**Authors:** 


**Atypic Typhoid.** Roger Voisin, Paris Medical, May 13, describes 5 groups of cases: 1. "Typhoidette," lasting 7-13 days, with Widal reaction constantly present, rose spots, ordinary symptoms but without diarrhoea or temperature above 39 C. 2. (17 cases) mainly ambulant cases, developing serious symptoms but lasting only 4-6 days after admission to hospital. 3. principal symptoms of gastric disturbance, temperature 38-38.5, initial vomiting and diarrhoea, relieved by purgation and diet of water followed by milk, but spleen remaining large for some time. 4. enterocolitis, constipation, marked symptoms of autointoxication, long duration. 5. cases marked by successive recrudescence or relapse with intervening apyrexia but symptoms rather mild.

(Note: Typhoid has been a very rare disease in Paris and probably in most French cities, for ten years or more. It is possible, therefore, that the suddenly acquired opportunities for observing numerous cases of the disease in military practice has impressed the author more strongly with atypic manifestations than would occur in the U. S. where typhoid has been very common till the last few years and is still frequent in numerous localities. Practically all the "groups" which he enumerates are encountered here. It may also be that previous unsuccessful—and perhaps partial—vaccination has really modified the course of the disease. In our own experience, we have been struck by the relative lack of serious results in ambulant cases).

